# Effectiveness of two physical therapy interventions, relative to dental treatment in individuals with bruxism: study protocol of a randomized clinical trial

**DOI:** 10.1186/1745-6215-15-8

**Published:** 2014-01-07

**Authors:** Cinthia Santos Miotto Amorim, Eliete Ferreira Osses Firsoff, Glauco Fioranelli Vieira, Jecilene Rosana Costa, Amélia Pasqual Marques

**Affiliations:** 1 Department of Physical Therapy, Speech and Occupational Therapy, School of Medicine, University of São Paulo, São Paulo, SP, Brazil; 2 Department of Operative Dentistry, School of Dentistry, University of São Paulo, São Paulo, SP, Brazil; 3 Department of Pediatric Otolaryngology, Federal University of São Paulo, São Paulo, SP, Brazil

**Keywords:** Bruxism, Physical therapy modalities, Massage, Muscle stretching exercises, Relaxation therapy, Imagination

## Abstract

**Background:**

Bruxism is a parafunctional habit characterized by grinding and/or clenching of the teeth. It may happen while awake (awake bruxism) or while sleeping (sleep bruxism). In adults, the prevalence is 20% for the awake bruxism and 8% for the sleep bruxism. Peripheral, central, and psychosocial factors influence the disorder, which may predispose to pain in the masticatory muscles and neck, headache, decreased pain thresholds in the masticatory and cervical muscles, limitation mandibular range of motion, sleep disorders, stress, anxiety, depression, and overall impairment of oral health. The aim of this study is to compare two distinct physical therapy interventions with dental treatment in pain, mandibular range of motion, sleep quality, anxiety, stress, depression, and oral health in individuals with bruxism.

**Methods/Design:**

Participants will be randomized into one of three groups: Group 1 (*n* = 24) intervention will consist of massage and stretching exercises; Group 2 (*n* = 24) will consist of relaxation and imagination therapies; and Group 3 (*n* = 24) will receive dental treatment. The evaluations will be performed at baseline, immediately after treatment, and at 2-month follow-up. Pain intensity will be assessed using the visual analogical scale, while pain thresholds will be determined using dolorimetry. Mandibular range of motion will be assessed using digital pachymeter. Sleep quality will be assessed by the Pittsburgh Sleep Quality Index, anxiety by the State*-*Trait Anxiety Inventory, stress by the Perceived Stress Scale-10, depression by the Beck Depression Inventory, and oral health will be assessed using the Oral Health Impact Profile - 14. Significance level will be determined at the 5% level.

**Discussion:**

This project describes the randomization method that will be used to compare two physical therapy interventions with dental treatment in the management of pain, mandibular range of motion, sleep quality, anxiety, stress, depression, and oral health in individuals with bruxism. The study will support the practice of evidence-based physical therapy for individuals with bruxism. Data will be published after study is completed.

**Trial registration:**

ClinicalTrials.gov, NCT01778881

## Background

The second edition of the International Classification of Sleep Disorders (ICSD-2) defines sleep bruxism as a stereotyped movement disorder characterized by grinding and/or clenching of teeth during sleep [1-5]. Awake bruxism is a semi-voluntary activity of teeth clenching that is rarely associated with audible sounds. The prevalence of awake bruxism is around 20%, while sleep bruxism affects around 8% of adults [4-6].

Peripheral, central, and psychosocial factors are relevant to the pathophysiology of bruxism [5,6], which may predispose to pain in the masticatory muscles and neck, headache, decreased pain thresholds in the masticatory and cervical muscles, limitation mandibular range of motion, sleep disorders, stress, anxiety, depression, and overall impairment of oral health. Bruxism is also associated with symptoms of other oral disorders [2,3,5].

Although some studies associate bruxism with craniofacial pain, the cause-effect relationship is not yet fully established [7-9]. Pain is not present in all individuals with bruxism, but studies show that repetitive activities related to parafunctional habits are considered important factors perpetuating pain [10]. Studies indicate that clenching and/or grinding of the teeth can trigger levels of muscle sensitization post exercise in the masticatory system, leading to damage of muscle fibers and surrounding tissues [8,11]. Currently the focus is on adaptation model of pain, which proposes that pain arises from muscle hyperactivity and, in turn, reduces muscle activity for the protection of the masticatory system [12].

Recent international consensus formed by a group of bruxism experts affirms the bruxism can be identified by anamnesis and physical examination, and confirmed with specific questionnaires [2,5], although there is also the polysomnography conducted in sleep laboratories [9,13].

Because bruxism is a multifactorial disorder, single specific treatments are not available, and multidisciplinary approaches administered by teams formed by dentists, physiotherapists, and other health professionals are often necessary [4-6]. Most treatment strategies are conservative, reversible, and symptomatic, aiming to prevent the consequences of the disorder [5,14,15].

Currently, physical therapy focuses mainly in two objectives: to decrease the adverse effects of bruxism to the masticatory system, and to increase self-awareness about this parafunctional habit. Techniques include therapeutic exercises, manual therapy, cognitive behavioral therapy, electrotherapy, acupuncture, postural awareness, and muscular awareness relaxation [15-23]. Dental treatments are often necessary, whose overall goal is to re-establish the occlusal harmony [4-6]. Among the several approaches, direct restoration of the dental surface using composite resin is a good option in order to re-establish incisal guides, promoting esthetic improvement and better function and occlusion [24,25].

In clinical practice, intra and extra oral massage of the masticatory muscles and massage of the cervical muscles are used and are associated with local circulation and metabolic improvements, as well as with decreasing muscle tonus [15]. Stretching exercises aim to increase mandibular range of motion, therefore decreasing pain and allowing for better mandibular rest positioning [16]. Relaxation therapies, such as progressive muscle relaxation, consist of a series of procedures involving implementation of controlled cycles of contraction and relaxation of different muscle groups. These techniques promote self-awareness of muscle tension and education on principles of muscle relaxation in activities that are relevant to normal life. Diaphragmatic breathing training and imagination (learning to create mental images of relaxed environments or activities) are relevant pieces of these therapies [18]. The literature is not clear regarding which physical therapy interventions are most effective for individuals with bruxism; therefore, more randomized controlled trials (RCTs) are necessary to clarify these questions. Moreover, little is known about the effect of two physical therapy interventions with dental treatment in individuals with bruxism.

Studies focusing physical therapy treatments to improve symptoms of bruxism such as massage of the masticatory and cervical muscles [15], stretching exercises [16], and relaxation therapies [18] are few and often do not describe in detail the treatment. Even rarer are studies that compare physical and dental therapies in individuals with bruxism [14,18]. Accordingly, our study adds to the evidence-based physical therapy practice addressing bruxism.

### Study aim

The aim of this study is to compare three treatments: two distinct physical therapy interventions (massage with stretching and relaxation and imagination therapies) with dental treatment in individuals with bruxism. In particular, we aimed to focus on pain, mandibular range of motion, sleep quality, levels of anxiety, stress, and depression, as well as on the overall status of oral health.

The hypothesis of this study is that the individuals with bruxism who receive one of physical therapy interventions (massage and stretching or relaxation and imagination therapies) will have greater reduction in pain intensity levels and better mandibular range of motion, sleep quality, levels of anxiety, stress and depression, and overall status of oral health compared to individuals who receive dental treatment as assessed immediately after the 6-week physical therapy intervention and that these benefits will be maintained until reassessment 2-month follow-up.

## Methods/Design

This study will be a RCT comparing two physical therapy interventions with dental treatment. Physical therapy will last 40 minutes and will happen twice a week for 6 weeks. Sessions will be conducted by the same physical therapist. Dental treatment will consist of two individual sessions conducted 1 week apart, each of it lasting 2 hours.

### Enrollment and eligibility criteria

The sample will consist of 72 patients, with established diagnosis of bruxism, that will be referred by Department of Dentistry at the School of Odontology, University of São Paulo.

#### Inclusion criteria

• Sleep Bruxism diagnosed according to the criteria of the International Classification for Sleep Disorders (ICSD) of the American Academy of Sleep Medicine (AASM) [1];

• Self-report of awake bruxism, documented by positive response to the question developed following the recommendations of Pintado [26];

• Aged between 18 to 60 years;

• A minimum pain intensity score of 3 on the Visual Analogical Scale [27].

#### Exclusion criteria

• More than two missing teeth, except third molars;

• Systemic and/or degenerative diseases;

• Arthrogenic or mixed temporomandibular disorder (TMD) according to the Research Diagnostic Criteria for Temporomandibular Disorders (RDC/TMD), axis I [28];

• Neurological or psychiatric diseases (with the exception of anxiety and depression);

• Using medications that influence sleep or motor behavior;

• Periodontal disorders;

• Abuse of alcohol and/or illicit drugs;

• Removable dentures, superior and/or inferior;

• Total dentures;

• Direct trauma or past surgery in the orofacial region;

• On physical, speech, dental, or psychological therapy at the time of study entry.

#### Procedures

All participants will be submitted to the same assessments, which will happen at baseline, immediately after treatment and at 2-month follow-up. Assessments will be conducted by a blinded researcher. Demographic and anthropometric data will be collected, as well as bruxism history. Examinations will also look for degree of severity of the temporomandibular disorder [29], bruxism [28,30,31], and pattern of cervical spine curvature [32]. Primary (pain and mandibular range of motion) and secondary (sleep, anxiety, stress, depression, and oral health) outcomes will be assessed.

### Randomization procedures

**Figure 1:**
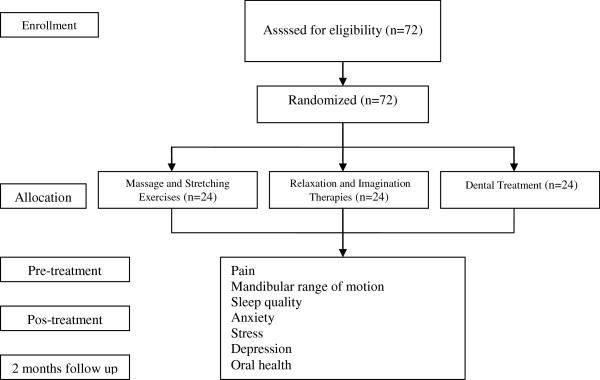
Flow diagram of the study.

Participants will be randomized into one of three possible groups: Group 1 (*n* = 24) intervention will consist of massage and stretching exercises; Group 2 (*n* = 24) will consist of relaxation and imagination therapies; and Group 3 (*n* = 24) will receive dental treatment, through a computer-generated randomization schedule that will be performed by an independent researcher, not involved in other study procedures. The allocation of participants will be concealed by using consecutive numbered, sealed, and opaque envelopes. The flow of the study is summarized in Figure 1.

## Outcome measures

### Primary outcomes

#### Pain

Pain intensity of the masseter, anterior temporalis, upper trapezius, and sternocleidomastoid muscles will be assessed using Visual Analogical Scales, constructed as a 10 cm ruler and clenching, muscular, and mental relaxation sensations and sleep difficulties will also be assessed using the same scale. On the left side of the scale, descriptors will be: no pain, no clenching, complete relaxation, and no difficulties falling sleep. On the right side, they will be: unbearable pain, maximum clenching, no relaxation, and being incapable of sleeping [27].

The pain threshold will be assessed using a dolorimeter with a rubber extremity with 1 cm of diameter. Perpendicular pressure on the skin will be applied on the trigger points of the upper trapezius, sternocleidomastoid, masseter, and anterior temporalis muscles, bilaterally. Participants will be seated straight, with feet touching the floor to the identification of the trigger points and demonstration of the dolorimeter technique in their forearm. After, patients will stay in dorsal decubitus with head discretely tilted to the opposite side of the assessments. Pain thresholds will be measured for each point, marked with a pencil. Lower thresholds are indicative of higher nociceptive sensation [33-35]. Pain thresholds will be considered positive when values are lower than 2.6 kg/cm^2^[33,36].

#### Mandibular range of motion

A digital pachymeter will be used in order to measure the mandibular range of motion (Digimess®) [36]. Reference points will be determined using the system proposed by Steenks and Wijer [37], using the maximal distance between the superior and inferior incisive teeth at the incisive line, with the participant seated, with aligned spine and feet touching the floor.

### Secondary outcomes

#### Quality of sleep

Quality of sleep during the last month will be assessed using the Pittsburgh Sleep Quality Index (PSQI). This index has been translated and validated into Portuguese. It consists of 19 questions grouped under seven domains: subjective sleep quality; sleep latency; sleep duration; usual sleep efficiency; sleep disturbances; use of medications; and diurnal dysfunction. Each domain is scored (0 to 3) and a total score (0 to 21) is calculated as follows: scores ranging from 0 to 4 are indicative of good sleep quality; scores from 5 to 10 suggest poor sleep quality; scores above 10 are suggestive of sleep disorders. Five other questions are answered by participants’ spouses or partners, and further characterize sleep quality [38,39].

#### Anxiety

Anxiety will be assessed using the State*-*Trait Anxiety Inventory (STAI), which has been translated and validated to the Portuguese language. It consists of two independent scales, with 20 questions each, measuring anxiety as a trait (in general) and as a state (at the moment). Each question is scored (from 1 to 4), and scores for the scales range from 20 to 80 as follows: mild anxiety (20 to 34); moderate anxiety (35 to 49); high anxiety (50 to 64); and very high anxiety (65 to 80) [40,41].

#### Stress

Stress will be assessed with the Perceived Stress Scale (PSS-10), which has also been translated and validated [42]. It consists of 10 questions with scores ranging from 0 to 4. Some questions (4, 5, 7 and 8) suggest absence of stress and have an inverted scoring system. Total scores range from 0 to 40, and higher values suggest higher stress levels [43].

#### Depression

The Beck Depression Inventory (BDI) will be used in order to assess depression. This inventory, which has been translated and validated into Portuguese, consists of 21 groups of four statements, assessing depressive symptoms over the last week, such as hopelessness, irritability, guilt, feelings of being punished, as well as physical symptoms such as fatigue, weight loss, decreased libido. Scores below 10 suggest lack or minimal depressive symptoms; from 10 to 18, mild to moderate depression; from 19 to 29, moderate to severe depression; and symptoms of 30 or more are suggestive of severe depression [40].

#### Oral health

Oral health will be assessed using the Oral Health Impact Profile - 14, a shortened version of the Oral Health Impact Profile - 49 [44]. The questionnaire has been translated and validated into Portuguese [45]. It consists of 14 questions with five response options: (0) never; (1) rarely; (2) sometimes; (3) often; and (4) always. Total scores range from 0 to 56, and higher scores are suggestive of poorer oral health [46].

#### Intervention

After the initial assessment, participants will receive information about bruxism, including its symptoms and consequences. They will also be educated about the study procedures. All participants will be submitted to the same assessments, at baseline, immediately after treatment, and at 2-month follow-up.

In that assessment at the 2-month follow-up, participants will receive educational material, with the purpose of providing important information about how to maintain the control of the bruxism after physical therapy interventions or dental treatment [15,16,47].

### Group 1: massage and stretching

The routine to be used in this group (stretching, massage, and diaphragmatic breathing) is described in Table 1[15,35,36,48-51].

**Table 1:** Description of the massage and stretching exercises

**Interventions**	**Description**	**Sets/duration**
Massage	Patient lying supine knees semi-flexed and head on pillow with proper height.	Inspiration/Pause/Expiration: 3:3:3 seconds, five sets
	Diaphragmatic breathing training.	
	**Extra-oral:** sliding, kneading, friction, and trigger points release (ischemic compression) with cream and emphasis on the masseter, anterior temporalis, and muscles of the neck as the upper trapezius and sternocleidomastoid, bilaterally.	Ischemic compression (maximum 60 seconds with 10-second rest between compressions), five sets
	**Intra oral:** sliding, trigger points release (ischemic compression), and circular and transverse friction, with glove, on the masticatory muscles, with emphasis on the masseter, bilaterally.	
Muscle stretching exercises	Patient lying supine knees semi-flexed and head on pillow.	Three sets of 30 seconds
	**Masticatory muscles:** Passive exercises with the help of the therapist (using gloves): elevators of the jaw (masseter, temporalis and medial pterygoid), retrusores (posterior temporal, digastric), lateral (contralateral lateral pterygoid) and circular movements of the jaw.	
		Intervals between sets of 10 seconds
	Patient sitting, feet flat on the ground and spine aligned.	
	**Muscles of the head and cervical spine:** Passive exercises with the help of the therapist: extensor (upper trapezius, levator scapulae, suboccipital), flexor (sternocleidomastoid and anterior scalene), lateral flexor (sternocleidomastoid, upper trapezius, middle and posterior scalenes, bilaterally), and rotators (upper trapezius and sternocleidomastoid contralateral), bilateral.	

### Group 2: relaxation and imagination therapies

The routines to be adopted will focus on progressive muscular relaxation associated to imagination and diaphragmatic breathing. They are described in Table 2[18,52-54].

**Table 2:** Description of the relaxation and imagination therapies

**Interventions**	**Description**	**Sets/duration**
Progressive muscle relaxation	Patient sitting with feet and head supported.	Inspiration/Pause/Expiration: 3:3:3 seconds, five sets
	Diaphragmatic breathing training.	
	First to third session: contraction and relaxation of the muscle groups: arm, forearm and hands; frontal, eyes and nose; mouth and jaw.	
	Fourth to sixth session: contraction and relaxation of the muscle groups: neck; shoulder; mouth and jaw.	
		Contraction/Relaxation: 10:20 seconds, two sets of each muscle group
	Seventh to ninth session: repeating sequence of previous sessions;	
	Tenth to 12th session: complete sequence of the relaxation.	
Imagination	Patient sitting with feet and head supported and closed eyes.	Three scenes (6 to 9 minutes each)
	Patient in relaxation (mostly jaws) and concentration in mental imagery of the scenes of your own, verbally guided by the therapist, with themes such as:	
	- Sunset	
	- Clouds	
	- Rivers	
	- Flowers	
	- Mountains	
	- Forests	
	- Beaches	
	- Other	

### Group 3: dental treatment

Participants in this group will be treated by a dentist, and will be submitted to the same assessments and education. Restoring treatment will involve direct reconstruction of the anterior guides (incisive faces of the incisive and canine teeth) with resin. Two individual sessions will be conducted 1 week apart, and each will last up to 2 hours. Patients will be reassessed 30, 60, and 90 days after treatment end.

### Sample size calculation

Sample size was defined in order to detect a 2-point difference between groups on the pain intensity outcome measured by the Visual Analogical Scales*,* assuming a standard deviation of 2 points. Power was defined as 80% for an alpha of 5% and attrition (drop-outs) of 20%. Accordingly, 24 participants per group will be needed.

#### Statistical analyses

Descriptive statistics will be used, and data will be summarized using mean, standard deviations, and percentiles. Normality of data distribution will be tested using the Kolmogorov-Smirnov test. Normal variables and comparison among three groups (massage with stretching, relaxation and imagination therapies, and dental treatment) will be assessed at baseline, immediately after treatment and at 2-month follow-up using Analysis of Variance. Post-hoc tests will consist of the Turkey test for parametric variables, and the Dunn’s test for non-parametric variables. Analyses will be conducted using SPSS 19 and SigmaStat 3.5. A level of significance of 5% will be used.

### Ethics and data security

This trial was approved by the Ethics Committee of the School of Medicine of the University of São Paulo (protocol study - 209369/2013). All patients will be asked to provide written, informed consent prior to randomization, using standard forms. This trial is registered in ClinicalTrials.gov (a service of U.S. National Institutes of Health) under the number NCT01778881.

## Discussion

The purpose of this randomized clinical trial is to compare two physical therapy interventions with dental treatment in the management of pain, mandibular range of motion, sleep quality, anxiety, stress, depression, and oral health in individuals with bruxism. The study will support the practice of evidence-based physical therapy for individuals with bruxism. Data will be published after the study is completed.

## Trials status

We are currently recruiting participants.

## Competing interests

The authors declare that have no competing interests.

## Authors’ contributions

CSMA, EFOF, GFV, JRC, and APM were responsible for the design of the study. APM will act as the study coordinators. All authors read and approved the final manuscript.
